# Potential Role of SdiA in Biofilm Formation and Drug Resistance in Avian Pathogenic *Escherichia coli*

**DOI:** 10.3390/ani14152199

**Published:** 2024-07-28

**Authors:** Haowen Hai, Mengyang Yang, Zhuo Cheng, Kai Ma, Fei Shang

**Affiliations:** School of Life Sciences, Anhui Agricultural University, Hefei 230036, China; haihaowen2024@163.com (H.H.); yangmy4321@163.com (M.Y.); chengzhuo725@163.com (Z.C.); makaiunique@163.com (K.M.)

**Keywords:** avian pathogenic *Escherichia coli*, SdiA, biofilm formation, drug resistance

## Abstract

**Simple Summary:**

Avian pathogenic *Escherichia coli* (APEC) constitutes a notable etiological agent of colibacillosis, an inflammatory disease in birds that leads to considerable economic losses within the poultry industry. In this study, we probed into the impact of the quorum sensing regulator SdiA on the transcriptional profiling of an APEC strain and proved the potential regulatory role of SdiA in biofilm formation, motility, and drug resistance. The discoveries disclose the involvement of SdiA in the pathogenesis of APEC and will furnish some novel insights into the prevention and control of colibacillosis induced by APEC.

**Abstract:**

Avian pathogenic *Escherichia coli* (APEC) constitutes a significant cause of colibacillosis, a localized or systemic inflammatory disorder in avian species, resulting in considerable economic losses within the global poultry industry. SdiA (suppressor of division inhibitor) is a transcription factor recognized as a LuxR homolog in *Escherichia coli*, regulating various behaviors, including biofilm formation, multidrug resistance, and the secretion of virulence factors. However, the function of SdiA in APEC strains and its correlation with virulence and multidrug resistance remains unknown. This study probed into the function of SdiA by analyzing the effect of *sdiA* deletion on the transcription profile of an APEC strain. The microarray data revealed that SdiA upregulates 160 genes and downregulates 59 genes, exerting a particularly remarkable influence on the transcription of multiple virulence genes. A series of antibiotic sensitivity tests, biofilm formation assays, motility assays, and transcriptome analyses were performed, while a Normality test and *t*-test were conducted on the datasets. This research confirmed that SdiA inhibits biofilm formation by 1.9-fold (*p*-value < 0.01) and motility by 1.5-fold (*p*-value < 0.01). RT-qPCR revealed that SdiA positively regulates multidrug resistance by upregulating the expression of *yafP*, *cbrA*, and *eamB*. Collectively, the results of this study indicate the role of SdiA in the pathogenesis of APEC by controlling biofilm formation, motility, and multidrug resistance.

## 1. Introduction

*Escherichia coli* is a bacterium widespread in animals, humans, and the environment. As an opportunistic pathogen, *E. coli* is one of the most important pathogenic agents in poultry, which significantly threatens the health of livestock and food safety and public safety [1]. In particular, avian pathogenic *E. coli* (APEC) has been considered to be a major cause of colibacillosis, a localized or systemic inflammatory disease including airsacculitis, perihepatitis, pericarditis, coliform cellulitis, swollen-head syndrome, and sometimes fatal septicemia [2,3,4,5]. Colibacillosis is responsible for significant economic losses in the poultry industry worldwide [6]. Therefore, the pathogenesis, the virulence determinants, and the invasion mechanisms by which APEC strains cause serious infections deserve more attention [7].

Bacteria can communicate with one another by producing and secreting signal molecules in the surrounding environment to coordinate gene expression across the bacterial community, and the mechanism is termed quorum sensing (QS). In Gram-negative bacteria, the most common QS system is the LuxR/LuxI-type system with acyl homoserine lactone (AHL) as the signaling molecule [8]. The AHL signals, also called AI-1, are produced by the LuxI synthase and detected by the cognate LuxR transcription factor. The LuxR/LuxI-type system was first discovered in *Vibrio fischeri* [9], and since then, more than 50 species containing LuxR/I homologs have been reported. However, *Salmonella enterica* and *E. coli* only encode a LuxR homolog, SdiA, but not the LuxI-type synthase. In *Salmonella* and *E. coli*, SdiA can detect the quorum-sensing signals, such as AHLs, indole, and AI-2 produced by other bacteria, and is associated with intraspecies and interspecies communication [10]. By binding and interacting with the extracellular signals, SdiA can regulate diverse social behaviors like cell division, motility, biofilm formation, chemotaxis, multidrug resistance, and virulence [11,12,13].

The biofilm formed by pathogens on surfaces is considered to be the primary mechanism of virulence associated with many infections and diseases in humans and animals [14]. The biofilm matrix is primarily constituted by autocrine polysaccharides, proteins, and nucleic acids, which enables bacteria to evade the host immune system, prevent the ingress of antibiotics, and develop antibiotic resistance [15]. Curli fimbriae, type one fimbriae, and motility have been described to be fundamental to the formation of a biofilm by *E. coli* strains [16,17]. Lee et al. demonstrated that SdiA decreases early *E. coli* biofilm formation by repressing motility and influencing acid resistance [18]. In enterohaemorrhagic *E. coli* O157:H7, SdiA appears to act as a strong repressor of genes encoding flagella and curli fimbriae and decreases the adherence to HEp-2 epithelial cells [19]. Deletion of *sdiA* in atypical enteropathogenic *E. coli* resulted in the formation of thicker biofilm structures and showed increased motility [20]. However, the role of SdiA in APEC has never been reported.

The spread of antibiotic-resistant bacteria and the infections caused by multidrug-resistant (MDR) bacteria have become crucial problems that attract increasing attention worldwide [21,22]. The growing occurrence of MDR in APEC is also of great concern to the poultry industry [23]. Potentiated sulfonamides are usually used to treat avian colibacillosis as first-choice antimicrobial agents, whereas tetracyclines and aminopenicillins (ampicillin and amoxicillin) are considered the second-choice [24,25]. Fluoroquinolones (FQ) have been widely used in MDR *E. coli* strains that produce extended-spectrum b-lactamases (ESBLs). Thus, quinolone resistance in APEC is an important problem. Overexpression of *sdiA* confers multidrug resistance, especially quinolone resistance, by positively regulating the multidrug resistance pump AcrAB in *E. coli* [26]. Whether SdiA has an effect on antibiotic resistance in APEC remains unknown.

In the present study, we evaluated the influence of *sdiA* deletion on the gene transcriptional profiling of an APEC strain and described the phenotypic changes arising from *sdiA* inactivation. The results showed the involvement of SdiA in biofilm formation, motility, and multidrug resistance, suggesting a connection between SdiA and the pathogenesis of the strain APEC40.

## 2. Materials and Methods

### 2.1. Bacterial Strains, Plasmids, and Culture Conditions

The strains and plasmids employed in the study are enumerated in Table 1. The APEC40 strain was isolated from a diseased pigeon, which has been shown to be pathogenic for chickens by the laboratory [27]. The strains were cultivated at 37 °C in Luria broth (LB) with continuous shaking at 200 rpm or on LB agar plates containing 1.5% agar. The growth of bacterial cells was monitored by measuring the absorbance at 600 nm with a spectrophotometer. When necessary, antibiotics were applied at the following final concentrations: 100 μg/mL ampicillin (Amp), 50 μg/mL kanamycin (Kan), or 30 μg/mL chlorampheicol (Cm).

### 2.2. General DNA Manipulation

Bacterial genomic DNA was extracted using a standard approach for Gram-negative bacteria. Plasmid DNA was prepared using a plasmid extraction kit (Promega, Madison, WI, USA) in accordance with the manufacturer’s instructions. The DNA fragments were amplified by polymerase chain reaction (PCR) with the use of Taq or PrimeSTAR^®^Max DNA Polymerase (Takara Bio Inc., Dalian, China). The amplified DNA fragment was purified from an agarose gel by using a gel purification kit (Promega, Beijing, China) as per the manufacturer’s instructions. The purified DNA fragments were digested with restriction enzymes (Takara, Dalian, Liaoning, China) and ligated by T4 DNA ligase (Takara) following standard methods. The primers for amplifying nucleotide sequences in this study were designed by means of Primer Premier 5.0 software (Primer Premier Software Inc., San Diego, CA, USA) and are listed in Table 2.

### 2.3. Construction of sdiA Deletion Mutant

The APEC40 mutant of *sdiA* deletion was generated through λ-Red Homologous Recombination [28]. The chloramphenicol resistance cassette (cat) from the PKD3 plasmid was amplified by PCR using two homologous arm primers, 40-SdiA-F and 40-SdiA-R. Subsequently, the *sdiA* target fragments were electrically transformed into APEC40 receptor cells in which the λ-Red recombinase was expressed by the PKD46 plasmid. After an electric shock, the material was immediately transferred into an EP tube containing 900 μL of nonresistant LB and resuscitated in a shaking incubator at 37 °C for 1 h. Then, the transfer material was spread on an LB plate containing 30 μg/mL of chloramphenicol for screening, and PCR amplification was carried out to verify the *sdiA* mutant using primers of Check-SDIA-F and Check-SDIA-R. The resulting *sdiA* mutant was inoculated in liquid LB containing 30 μL/mL of chloramphenicol and placed in a shaking incubator at 42 °C overnight to remove the PKD46 plasmid, and then the Pcp20 plasmid was transferred to the receptive mutant for cat knockout. Finally, PCR amplification was performed to verify the APEC40 mutant of *sdiA* deletion with primers Check-SdiA-F and Check-SdiA-R, and was then further confirmed by DNA sequencing. The mutant strain was named as APEC40/Δ*sdiA*.

### 2.4. Construction of the Complementary Strain

The *sdiA* gene and its promoter region were amplified from the chromosomal DNA of the APEC40 strain by PCR using the primers pC-*sdiA*-F and pC-*sdiA*-R. The resulting products were purified using the PCR Purification Kit from the whole genome of APEC40 and cloned into the low-copy plasmid pSTV28 (Takara) through enzyme digestion and DNA ligation. Subsequently, the recombinant plasmids pC*sdiA* were transformed into *E. coli* DH5a chemically competent cells. Positive colonies were selected by incubation on LB agar plates containing 30 mg/mL of chloramphenicol and further confirmed through DNA sequencing. The recombinant plasmids pC*sdiA* were extracted, purified, and transformed into the *sdiA* mutant strain to obtain the complementary strain APEC40Δ*sdiA*/pC*sdiA*.

### 2.5. Bacterial Growth Curves

After overnight culturing, the strains APEC40, APEC40/Δ*sdiA*, and the complementary strain were inoculated into fresh liquid LB medium and diluted to a specific concentration (OD600 = 0.03). The diluted bacteria were then incubated in a shaking incubator at 37 °C for 24 h, during which the bacterial density was measured every 2 h using a spectrophotometer under UV light at 600 nm. A bacterial growth curve was plotted by mapping the OD values at 600 nm against time.

### 2.6. Biofilm Formation Assays

The assays for biofilm formation were performed according to a method adapted from a previous study [29]. The overnight cultures of bacteria were diluted with 3 mL of fresh LB medium to an OD600 of 0.3. After incubation at 37 °C for 20 h, the medium was discarded, and the tubes were rinsed three times with sterile phosphate-buffered saline (PBS) and air-dried. The biofilm formation cells were fixed with 100% methanol for 5 min, washed, and stained for 20 min with 0.1% (*w*/*v*) crystal violet (CV) (Sangon, Shanghai, China). After washing and drying, the adsorbed dye was dissolved with 33% glacial acetic acid (Sangon, Shanghai, China). Subsequently, the absorbance was measured at 492 nm using a MicroELISA Autoreader (Thermo Scientific, Pittsburgh, PA, USA) in single-wavelength mode to quantitatively detect the biofilm. The experiment was independently repeated three times.

### 2.7. Motility Tests

Motility assays were performed as described by Kim et al. [30]. Bacterial cells of the strain APEC40 and the APEC40/Δ*sdiA* were cultivated in LB medium overnight and subsequently transferred onto the LB agar plates and incubated for 18 h at 37 °C. The individual fresh colonies were selected using a bacteriological needle and punctured into the motility agar plates (0.3% agar, 1% tryptone, and 0.25% NaCl) and then incubated at 37 °C. The diameters of the motility halo were measured and compared after 8 h of incubation.

### 2.8. RNA-seq, Library Generation, and Transcriptome Analysis

For the RNA-seq assays’ preparation, the overnight cultures of the strain APEC40 and the APEC40/Δ*sdiA* were diluted with fresh LB medium to an OD600 of 0.03, and then cultivated until reaching the late exponential phase. The cells were collected via centrifugation and resuspended in RNase-free water. Subsequently, the total RNA of the cells was extracted using the Trizol reagent (Transgen, Beijing, China). Further cDNA synthesis, fragmentation, and hybridizations were conducted by Biozeron Biotechnology Co. Ltd. (Jiading, Shanghai, China). After a series of steps, including cDNA synthesis, end repair, A-base addition, and ligation of the Illumina-indexed adaptors, libraries for sequencing were constructed using the VAHTS mRNA-seq v2 Library Prep Kit for Illumina^®^ (Vazyme, Piscataway, NJ, USA). The libraries were sequenced on the Illumina HiSeq 2500 platform.

For GO enrichment analysis, differentially expressed genes (DEGs) between APEC40 and the *sdiA* mutant strain were identified by using a false discovery rate *p*-value < 0.05 and log2 (fold-change) > 1.5 as cutoffs on the Gene Ontology Consortium website (geneontology.org, accessed on 19 April 2024). The Kyoto Encyclopedia of Genes and Genomes (KEGG) database (http://www.genome.jp/kegg, accessed on 20 April 2024) was utilized to analyze the functional enrichment of the differentially expressed genes.

### 2.9. RNA Isolation, cDNA Synthesis, and Quantitative Real-Time PCR Analysis

The overnight cultures of bacterial cells were diluted with fresh LB medium to an OD600 of 0.03 and then transferred into the 24-well plates (Corning, NY, USA) for growth to the late exponential phase. Subsequently, the cells were collected by centrifugation and resuspended in RNase-free water containing 10 mg/mL of lysozyme and 40 µg/mL of lysostaphin (both from Sangon, Shanghai, China). After incubation at 37 °C for 5 min, the total RNA of the cells was extracted using the Trizol reagent (Transgen, Beijing, China). The real-time PCR assays were conducted by using a PrimeScript 1st Strand cDNA synthesis kit (TaKaRa, Kusatsu, Shiga Prefecture, Kusatsu, Japan) and a StepOne real-time PCR system (Applied Biosystems, Foster City, CA, USA). All the transcription assays were repeated at least three times.

### 2.10. Drug Susceptibility Assays

The antibiotic susceptibility assays were carried out in accordance with the standards of the Clinical and Laboratory Standards Institute. The overnight cultures of the strain APEC40 and the *sdiA* mutant were diluted with fresh LB medium to an OD600 of 0.03. Doxycycline, gentamycin, ofloxacin, ciprofloxacin, kanamycin, and erythromycin were added to the LB medium at concentrations of 10 mg/mL, 3.2 mg/mL, 2.5 mg/mL, 2.5 mg/mL, 50 mg/mL, and 5 mg/mL, and then serially two-fold diluted. The bacterial cells were cultivated in the 96-well plates (Costar, Corning, Steuben, NY, USA) for 24 h at 37 °C. The minimum concentration of antibiotics capable of completely inhibiting the growth of bacterial cells was determined as the minimal inhibitory concentration (MIC); the final concentrations are listed in Table 3. All the susceptibility assays were repeated at least three times.

The CFU assays were performed as described previously, and as follows [31]. The overnight cultures of the strain APEC40, APEC40/Δ*sdiA*, and 40Δ*sdiA*/pC*sdiA* were diluted to a specific concentration (OD600 = 0.03) and shaken in a 37 °C shaking tank for 2 h. Subsequently, doxycycline, gentamicin, ofloxacin, ciprofloxacin, kanamycin, and erythromycin were added to the culture medium at concentrations of 3.125 mg/mL, 0.25 mg/mL, 1.56 mg/mL, 1.56 mg/mL, 7.81 mg/mL, and 28.4 mg/mL (1/2 MIC), respectively, and the shaking was continued for 3 h. Then, 0.1 mL of the cultures was successively transferred into 10 EP tubes containing 0.9 mL of LB for gradient dilution. Three aliquots (0.1 mL) of appropriate volumes were spread on LB AGAR plates and incubated at 37 °C overnight for colony counting. This experiment was repeated three times for each antibiotic.

### 2.11. Statistical Analysis

The data were analyzed by means of SPSS software version 15 (IBM Corp., Armonk, NY, USA) using a one-way ANOVA. The statistical significance of the differences among the groups was assessed using a Normality test and two-sample *t*-test, and a *p* value of less than 0.05 was defined as a statistically significant result.

## 3. Results

### 3.1. Influence of sdiA Deletion on Bacterial Growth

**Figure 1:**
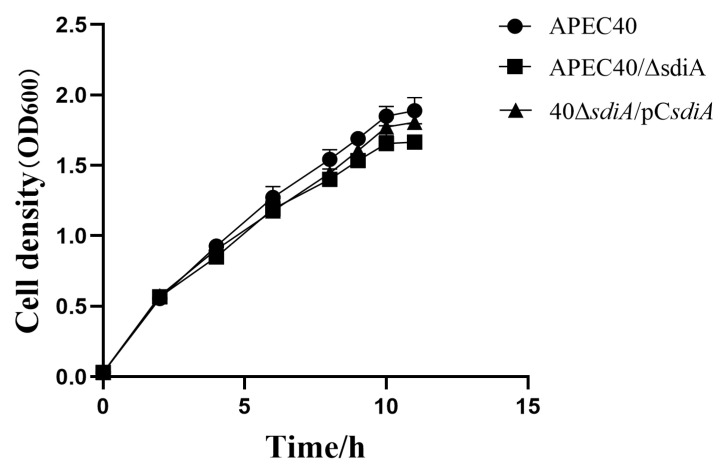
Growth curves of the APEC40, APEC/Δ*sdiA*, 40Δ*sdiA*/pC*sdiA* strains.

To validate the influence of *sdiA* deletion on the growth of the APEC40 strain, the growth curves of each strain were concurrently measured and contrasted under the identical culture condition (Figure 1). The outcome demonstrated that the growth rates of the APEC40, APEC40/Δ*sdiA*, and 40Δ*sdiA*/pC*sdiA* strains were comparable in the medium with the same LB, and there was no remarkable disparity in the exponential phase, stationary phase, and lag phase of the growth curve, suggesting that the deletion of *sdiA* has no impact on the growth of the APEC40 strain.

### 3.2. Transcriptional Profiling Analysis of the Strains APEC40 and APEC40/ΔsdiA

**Figure 2:**
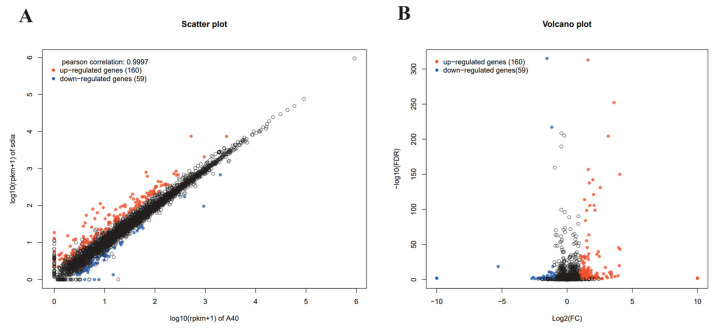
Differential expression analysis of APEC40 and APEC40/Δ*sdiA*. (**A**) Scatter plot of gene expression difference. Mean rpkm: used to measure the relative expression of the same gene in two different strain samples. The vertical axis and horizontal axis correspond to the logarithmic values of APEC40/Δ*sdiA* (*sdiA*) and APEC40 (A40) samples, respectively. Each point represents a gene. (**B**) In the volcano map of gene expression differences, the logarithmic values of difference multiple and error rates were, respectively, taken for the differential genes. The greater the absolute value of the horizontal axis corresponding to the points, the greater the gene expression difference; the smaller the value of the corresponding vertical axis, the lower the error rate.

**Figure 3:**
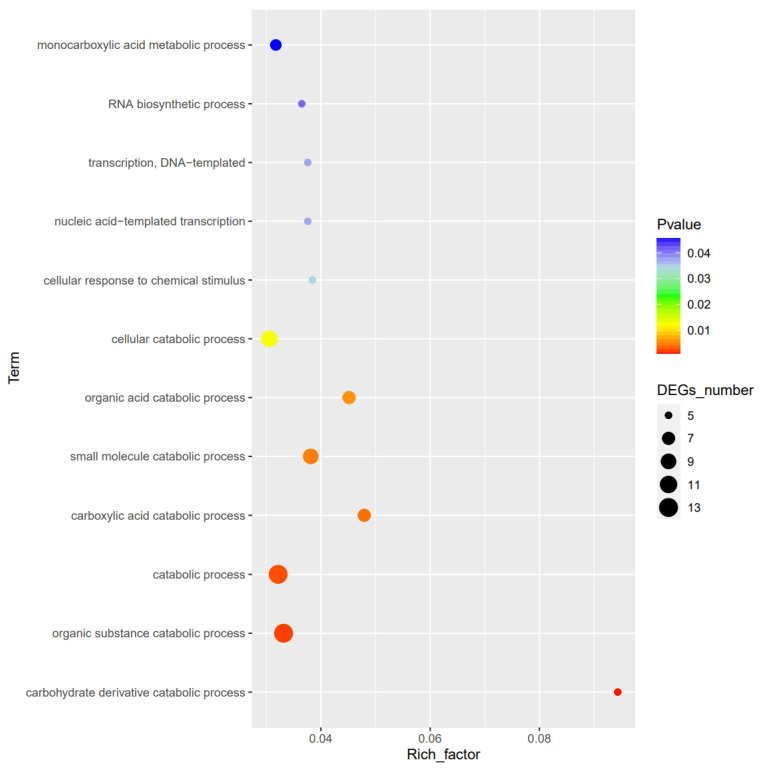
GO enrichment analysis of differential gene function.

**Figure 4:**
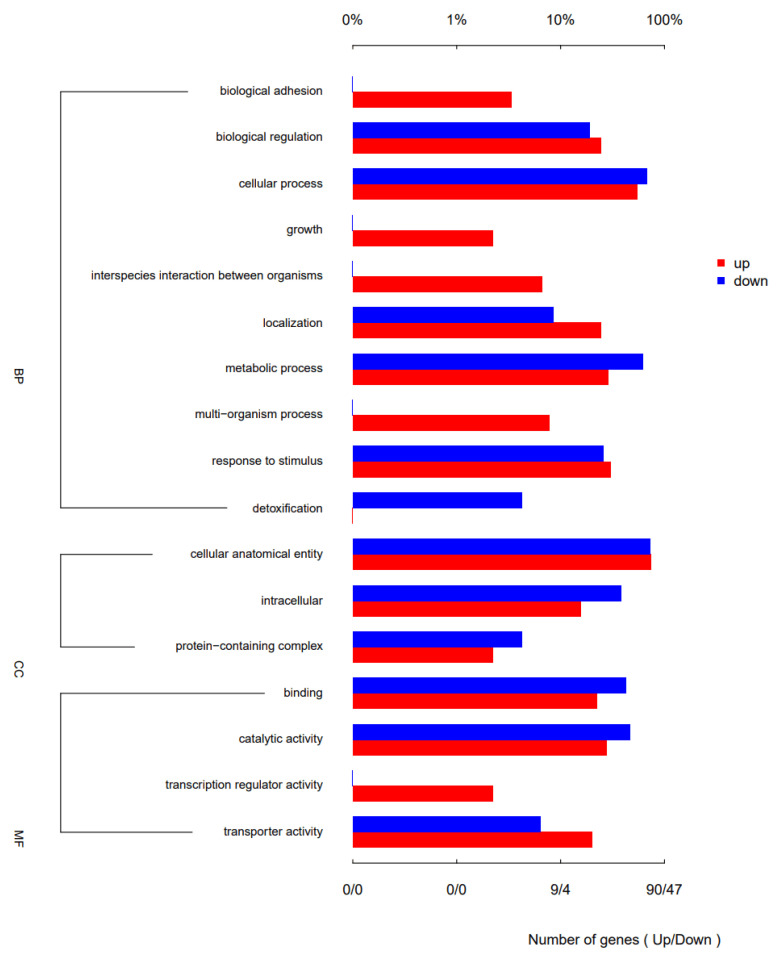
GO enrichment annotation of differential gene function.

To gain a comprehensive understanding of the function of SdiA in APEC, the transcriptional profiles were analyzed and compared between APEC40 and the corresponding *sdiA* mutant strain APEC40Δ*sdiA*. Based on the data of differential expression analysis, among a total of 4824 analyzed transcripts, 219 were identified as differentially expressed genes (DEGs) with a fold-change > 2 and *p* < 0.05. Among them, 160 genes were up-regulated, and 59 genes were down-regulated (Figure 2). The potential biological functions associated with the DEGs were identified through Gene Ontology (GO) analysis. The DEGs were classified into three major functional categories: biological process, cell component, and molecular function. As depicted in Figure 3 and Figure 4, the main biological processes in which the DEGs participated included carbohydrate catabolism (five genes), organic catabolism (18 genes), small molecule metabolism (22 genes), carboxylic acid catabolism (14 genes) (*p* < 0.01), cellular catabolism (11 genes), response to chemical stimuli (five genes), nucleic acid template transcription (six genes), and RNA biosynthesis (five genes) (*p* < 0.05), suggesting that the *sdiA* gene plays a crucial role in bioregulation, bioadhesion, cell growth, cell recognition, cell detoxification, taxis, and transporter activity.

**Figure 5:**
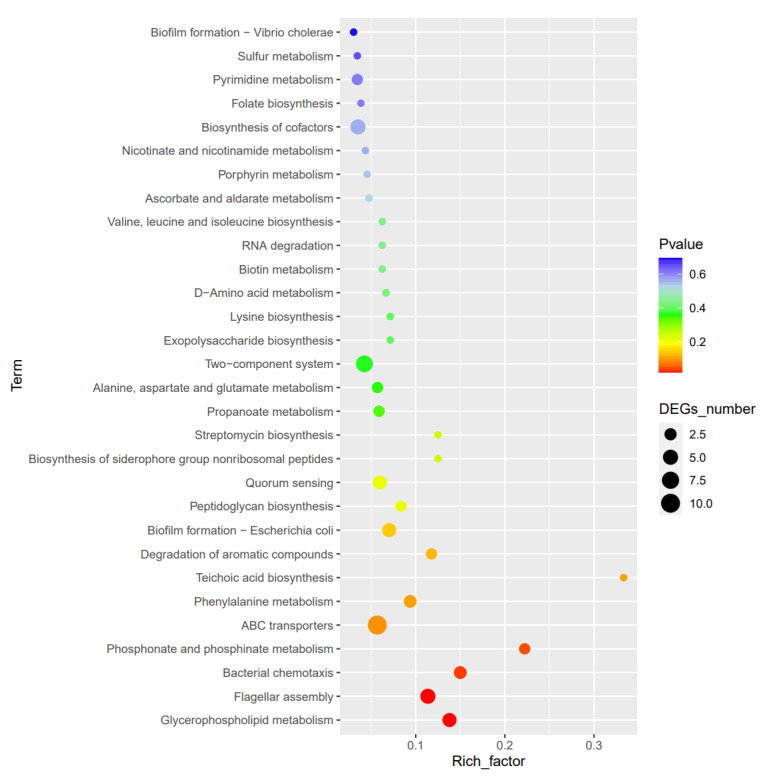
KEGG enrichment analysis of differential gene function.

To identify the active biological pathways influenced by SdiA, the DEGs were mapped to the KEGG database. As shown in Figure 5, the ten most frequently represented pathways were lipid metabolism (five genes), flagellar assembly (seven genes), bacterial taxis (five genes), biofilm formation (five genes), ABC transport system (seven genes), bi-component system (12 genes) (*p* < 0.2), polysaccharide biosynthesis (four genes), major metabolic pathways of biochemical molecules (12 genes), and biotin and cofactor synthesis (13 genes) (*p* < 0.5).

APEC possesses diverse virulence and pathogenesis factors or mechanisms that lead to colibacillosis in poultry. The microarray data revealed that the toxin expression gene *hlyE* was upregulated by 1.6-fold, *cdtB* was upregulated by 1.2-fold (*p*-value < 0.01), the adhesion expression genes *yadV*, *yfcV*, and *fimE* were upregulated by 3-fold, 2.6-fold, and 1.7-fold (*p*-value < 0.001), respectively, and the genes related to the iron acquisition systems *fepG* and *feoA* were upregulated by 4-fold and 1.2-fold (*p*-value < 0.01), while *phnO* associated with cell detoxification was downregulated by 2-fold (*p*-value < 0.01).

By analyzing the specific processes, functions, and pathways associated with SdiA, the annotations provide valuable information for a thorough understanding of the biological function of SdiA in APEC. According to the data, the deletion of *sdiA* significantly down-regulated the expression levels of several cell detoxification genes and up-regulated genes related to cell adhesion and flagella synthesis. The results indicate that SdiA might play an important role in multidrug resistance and biofilm formation.

### 3.3. Effects of sdiA Inactivation on the Biofilm Formation of the Strain APEC40

**Figure 6:**
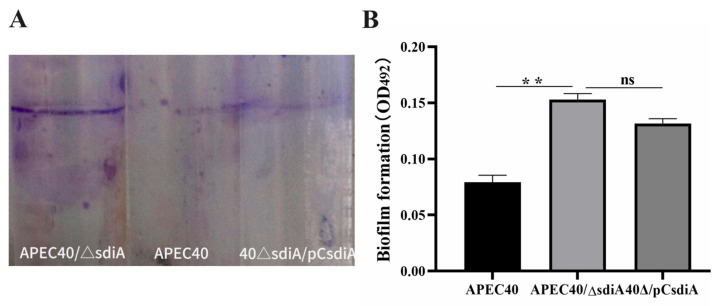
(**A**) Image of biofilm formation on the wall of a polystyrene tube. (**B**) Biomass measured at 492 nm after 12 h culturing, 0.1% crystal violet staining, and 33% glacial acetic acid dissolving. Error bars indicate SD; ** represents *p* < 0.01, and ns represents no significant difference.

SdiA has been reported to be a regulator of biofilm formation in many pathogens, but whether it is involved in biofilm formation in APEC remains unclear. To determine the role of SdiA in biofilm formation in APEC, the ability to form a biofilm between the APEC strain and the *sdiA* mutant strain APEC40/Δ*sdiA* was compared. As shown in Figure 6A, the *sdiA* mutant exhibited a significant increase in biofilm formation capacity in polystyrene tubes in comparison to the wild-type strain. Moreover, the quantity of biofilm was measured using a MicroELISA autoreader at wavelength 492 nm, and the results showed that the density of biofilm formed by the *sdiA* mutant was obviously higher than that of the wild-type. The complementary strain restored the phenotype Figure 6B. These results, which were assessed using a Normality test and two-sample *t*-test, indicate that SdiA negatively regulates biofilm formation in the strain APEC40.

### 3.4. Influence of sdiA Deletion on Motility of the Strain APEC40

**Figure 7:**
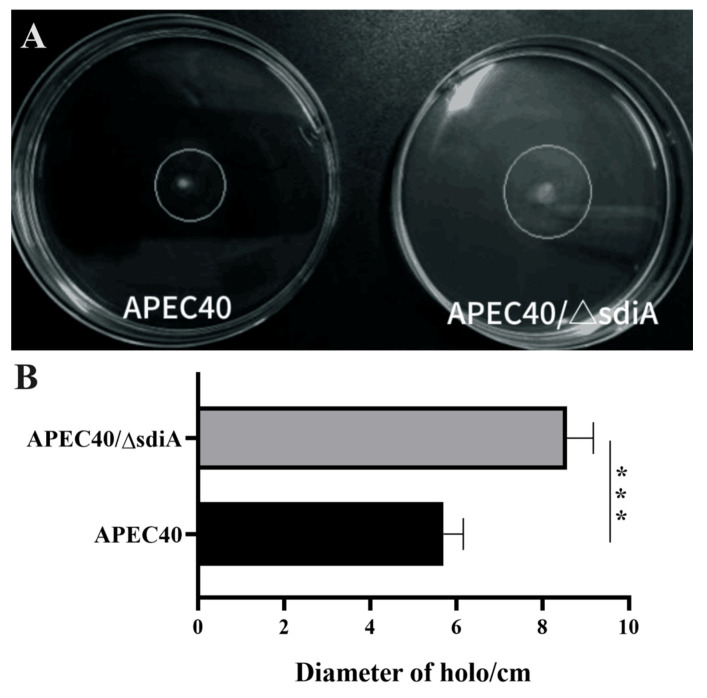
(**A**) Motility from APEC40 and APEC40/Δ*sdiA* strains after 8 h of incubation at 37 °C. (**B**) The mean diameter of the halo formed by APEC40 and APEC40/Δ*sdiA*. Error bars indicate SD; *** represents *p* < 0.001.

Since motility has been demonstrated to positively influence biofilm formation in *E. coli*, and our data revealed the relationship between SdiA and biofilm formation of strain APEC40, we speculated that motility can also be affected by SdiA. To investigate whether *sdiA* inactivation alters the motility capacity of the strain APEC40, the halo diameter between the *sdiA* mutant and the wild-type strain was compared by performing the motility assays. As shown in Figure 7A,B, the mean diameter of the halo on a semi-solid agar plate formed by the *sdiA* mutant had a significant increase compared to the wild-type strain. These results, assessed using a Normality test and two-sample *t*-test, suggest that SdiA might inhibit biofilm formation by affecting the flagellar motility of the APEC strain.

### 3.5. Inactivation of sdiA Changes Multidrug Resistance of the Strain APEC40

SdiA has been verified to serve as a QS regulator of a multidrug efflux transporter and exert an impact on the resistance of *E. coli* to a variety of antibiotics. Additionally, the microarray data indicated that the expression of several cell detoxification genes was modified by the *sdiA* mutation. To explore the influence of SdiA on the multidrug resistance of the strain APEC40, MIC assays were conducted by utilizing doxycycline, gentamicin, ofloxacin, ciprofloxacin, kanamycin, and erythromycin. The outcomes demonstrated that the MIC values of the six antibiotics were comparable in the APEC40 and APEC40/Δ*sdiA* strains. Nevertheless, at a one/two MIC, the two strains treated with doxycycline, gentamicin, and erythromycin exhibited significant disparities in the turbidity of the bacterial fluid.

**Figure 8:**
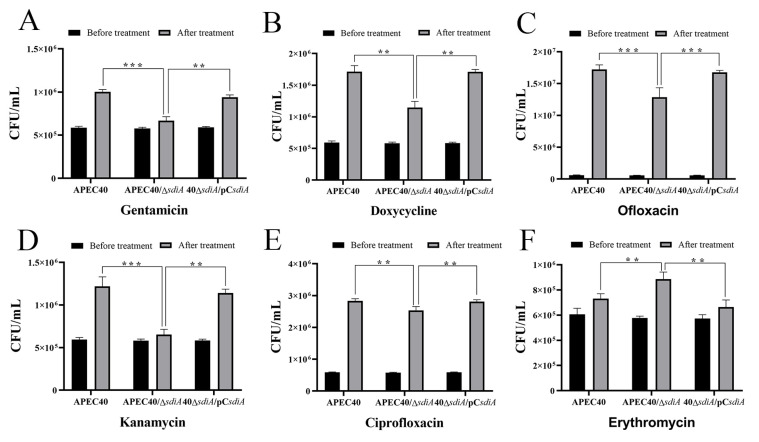
CFU assays of the APEC40, APEC40/Δ*sdiA*, and 40Δ*sdiA*/pC*sdiA* in the presence of six different antibiotics: (**A**) Gentamicin, (**B**) Doxycycline, (**C**) Ofloxacin, (**D**) Kanamycin, (**E**) Ciprofloxacin, and (**F**) Erythromycin. Error bars indicate SD; ** represents *p* < 0.01, *** represents *p* < 0.001.

To further validate the effect of SdiA on antibiotic resistance in the APEC40 strain, the CFU assays were executed to compare the survival rates of the wild-type, *sdiA* mutant, and complementary strain cells grown in LB supplemented with the aforementioned antibiotics. The results inspected using a Normality test and two-sample *t*-test revealed that the *sdiA* deletion mutant displayed decreased survival rates in the presence of antibiotics such as doxycycline, gentamicin, ofloxacin, ciprofloxacin, and kanamycin, suggesting that the deletion of *sdiA* downregulated the bacterial resistance to these antibiotics. As depicted in Figure 8, the *sdiA* mutants exhibited the most pronounced decline in survival compared to the wild-type in the presence of ofloxacin (C) and doxycycline (B), followed by the presence of kanamycin (D) and gentamicin (A). The difference was minimal in the presence of ciprofloxacin (E). These data suggested a positive effect of SdiA on the multidrug resistance of the APEC strain. However, conversely, we discovered that the survival rates of APEC40 strains in the presence of erythromycin were augmented after the deletion of *sdiA* (F).

### 3.6. The Deletion of sdiA Modifies the Expression of Virulence and Drug Resistance Genes

**Figure 9:**
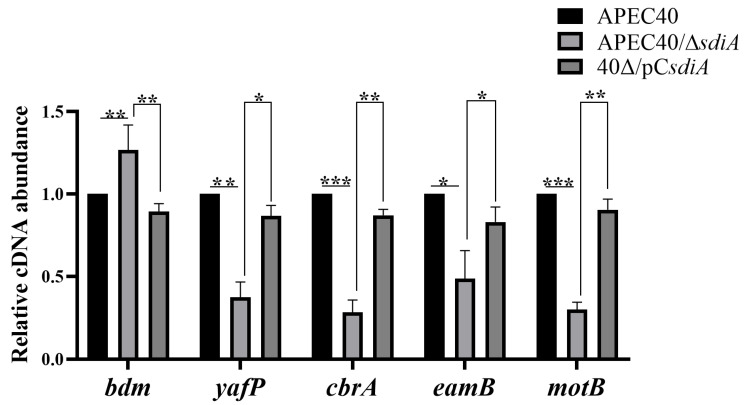
Transcriptional levels (cDNA abundance) of genes associated with drug sensitivity and biofilm formation were measured. RT-qPCR was used to determine the relative transcription levels of target genes in APEC40, APEC40/Δ*sdiA,* and 40Δ*sdiA*/pC*sdiA*. Error bars indicate SD, * represents *p* < 0.05, ** represents *p* < 0.01, *** represents *p* < 0.001.

To explore how SdiA regulates bacterial susceptibility to antibiotics and biofilm formation, we carried out RT-qPCR experiments to assess the transcriptions of genes associated with biofilm formation and drug sensitivity as recommended in the transcriptome analysis, such as *bdm*, *yafP*, *cbrA*, *eamB*, and *motB*. The results examined using the Normality test and two-sample *t*-test demonstrated that, in contrast to the wild-type strain APEC40, the *sdiA* deletion strain exhibited a 1.53-fold upregulation in the transcription level of *bdm* (*p*-value < 0.01), a gene related to biofilm formation, and a down-regulation in the transcription levels of drug resistance-related genes *yafP*, *cbrA*, *eamB*, and *motB* by 2.68-fold (*p*-value < 0.01), 3.53-fold (*p*-value < 0.001), 2.4-fold (*p*-value < 0.05), and 3.3-fold (*p*-value < 0.001), respectively. The complementary strain restored the transcription levels of these genes (Figure 9). These results indicate that the Rcs signal-induced increase in *bdm* transcription levels enables the mutant strain to form biofilms more effectively [32], and SdiA affects the antibiotic sensitivity of APEC40 by regulating genes related to drug transport proteins.

## 4. Discussion

APEC strains belong to extraintestinal strain and are mostly associated with respiratory tract or systemic infections in birds, collectively known as colibacillosis, which causes severe economic losses in poultry industries. The strong pathogenicity of APEC is facilitated by the ability to form a biofilm, antibiotic resistance, and virulence factors, which are coded by virulence genes. Although the pathogenic mechanism of *E. coli* has been intensively studied for the last few decades, there remains a significant deficiency in our understanding of APEC, which requires much more detailed exploration. This study focuses on the pathogenic mechanisms related to the biofilm formation and drug resistance of APEC, hoping to provide some valuable insights into the prevention and control of APEC infections.

During the pathogenic process, the bacterial cells communicate with each other through quorum-sensing mechanisms, which enables bacteria to coordinate their behaviors to persistently survive and colonize in host environments. It has been reported that SdiA participates in controlling multiple key virulence traits, such as pili production, adhesion, motility, biofilm formation, multidrug resistance, and acid tolerance [33]. Nevertheless, the function of this QS regulator in APEC remains unknown. In the present study, we described the role of SdiA in virulence regulation, as well as the underlying mechanisms by which SdiA affects pathogenesis in an APEC strain.

APEC possesses diverse virulence and pathogenesis factors or mechanisms that lead to colibacillosis in poultry. These factors encompass but are not limited to adhesins, invasins, protectins, iron acquisition systems, toxins, two-component systems, a quorum-sensing (QS) system, transcriptional regulators, secretion systems, and genes associated with metabolism [34]. The microarray data revealed that the toxin expression gene *hlyE* was upregulated by 1.6-fold, *cdtB* was upregulated by 1.2-fold (*p*-value < 0.01), and the adhesion expression genes *yadV*, *yfcV*, and *fimE* were upregulated by 3-fold, 2.6-fold, and 1.7-fold (*p*-value < 0.001), respectively. Then, the genes related to the iron acquisition systems *fepG* and *feoA* were upregulated by 4-fold and 1.2-fold (*p*-value < 0.01), while *phnO* associated with cell detoxification was downregulated by 2-fold (*p*-value < 0.01).

Biofilm formation is an important virulence factor involved in the adhesion to and invasion of host cells. SdiA has been confirmed to be associated with biofilm formation in diverse bacterial species. Lee et al. showed that the inactivation of *sdiA* enhanced cellular adherence by upregulating the expression of the genes involved in the biosynthesis of flagella and fimbriae [18]. Cheng et al. demonstrated that *sdiA* represses the biofilm formation ability of *Cronobacter sakazakii* by diminishing its motility and adhesion [35]. Moreover, in *Klebsiella pneumoniae*, the *sdiA* mutant strain exhibited increased biofilm formation ability, correlating with the increased expression of type-1 fimbriae and bacterial cell adherence and aggregation [36]. Consistent with these reports, the results obtained in our research indicated that the deletion of *sdiA* enhanced the motility and biofilm formation capacity of the APEC40 strain by 1.5-fold (*p*-value < 0.01) and 1.9-fold (*p*-value < 0.01). The microarray data indicated that the flagellar motility regulation gene *motB* was downregulated by 2-fold (*p* < 0.001), the flagellar protein TfaQ was downregulated by 1.8-fold (*p* < 0.001), and *fld* was downregulated by 1.5-fold (*p* < 0.001). The SdiA mutant strain truly exhibited higher motility, mycelium expression, and biofilm formation ability, and this study verified the negative impacts of SdiA on biofilm formation and motility.

A previous study showed that the QS system is involved in the regulation of multidrug resistance by sensing stress signals [37]. In fact, SdiA participates in regulating the expression of multidrug transporters. Rahmati et al. established a connection between quorum-sensing and multidrug efflux and discovered that the overexpression of *sdiA* resulted in the enhanced expression of the drug transporter AcrAB, thereby increasing resistance to various antibiotics, such as chloramphenicol, kanamycin, tetracycline, and streptomycin [26]. The microarray data of this study indicated that the deletion of *sdiA* decreased the transcription levels of the drug efflux ABC transport system and the MFS transporter NimT by 1.2-fold (*p*-value < 0.01). RT-qPCR revealed that the cysteine output gene eamB, which is associated with drug transport, was also downregulated by 2.4-fold (*p* < 0.05), which might cause the increased sensitivity of the *sdiA* mutant to antibiotics. The antibiotic sensitivity assays demonstrated that the deletion of *sdiA* would indeed enhance the sensitivity of APEC40 to doxycycline, gentamicin, ofloxacin, ciprofloxacin, and kanamycin to different extents, which was similar to the findings obtained by Wael A. H. Hegazy et al. regarding the sensitivity of *sdiA* deficiency to antibiotics in *Salmonella enterica* [38].

Besides influencing the drug efflux system, *sdiA* can also respond to antibiotics by modifying the detoxification capacity, beta-lactamase hydrolase activity, and outer and inner membrane proteins of bacterial cells [39]. Transcriptomic findings demonstrated that the deletion of *sdiA* in the APEC40 significantly downregulated the expression of genes related to cellular detoxification, such as *phnO*, *decR*, and *yafP*. Meanwhile, the transcriptional levels of glycosyltransferase and phosphorylase genes were also decreased by 2-fold (*p*-value < 0.01). The reduction in cell detoxification ability might enhance the sensitivity of APEC40 to antibiotics, and the decline in ribonucleoside hydrolase activity would prevent bacteria from degrading nucleic acid molecules bound to antibiotics, thereby reducing drug resistance, which is in line with the results of C Petersen et al. [40]. Additionally, we have concentrated on *cbrA*, a gene capable of mediating drug resistance in *Escherichia coli* by modifying peptidoglycan precursors. CbrA is also reported to be a global regulator in *Pseudomonas aeruginosa* and is involved in resistance to various clinical antibiotics, including polymyxin B, ciprofloxacin, and tobramycin [41]. We utilized RT-qPCR to verify that the absence of *sdiA* indeed decreased the expression levels of *cbrA* and *yafP* by 3.35-fold (*p*-value < 0.01) and 2.68-fold (*p*-value < 0.01), respectively. The drug sensitivity assays indicated that the inactivation of *sdiA* made the APEC40 strain resistant to most antibiotics employed in the experiments. Overall, these outcomes imply that there is a notable association between SdiA in the strain APEC40 and antibiotic resistance.

However, our results showed that deletion of *sdiA* increased the bacterial resistance to erythromycin. It has been reported that the murein hydrolase mutant of *E. coli* showed higher levels of susceptibility to erythromycin, suggesting that murein hydrolase negatively regulates antibiotic sensitivity and the overexpression of murein hydrolase might increase resistance to erythromycin [42]. The microarray assays showed that the transcription levels of *mltD*, the murein transglycosylase D encoding gene, were significantly upregulated by *sdiA* deletion. These results can explain why the deletion of *sdiA* in the strain APEC40 resulted in a decrease in susceptibility to erythromycin.

To conclude, the results of this study indicated the role of the QS regulator SdiA in the pathogenesis of the strain APEC40. The *sdiA* deletion mutant showed upregulated biofilm formation ability and motility but downregulated multidrug resistance compared to the wild-type strain. In addition, microarray assays help us to comprehensively understand the function of SdiA in APEC strains. Real-time PCR data confirmed that SdiA affects multidrug resistance by regulating the transcriptions of *cbrA*, *yafP*, *eamB*, and *motB*, the transporter-encoding genes in *E. coli*. Since there are dozens of putative drug transporters in the *E. coli* genome, the precise mechanisms by which SdiA affects multidrug resistance still need further exploration.

## 5. Conclusions

The findings of this study demonstrated the involvement of the QS regulator SdiA in the pathogenesis of the APEC40 strain. The *sdiA* deletion mutant exhibited an increased ability in biofilm formation and motility while showing a decreased level of multidrug resistance compared to the wild-type strain. Furthermore, microarray assays were employed to gain a comprehensive understanding of SdiA’s function in APEC strains. Real-time PCR data confirmed that SdiA influenced multidrug resistance by regulating the transcription of *cbrA*, *yafP*, *eamB*, and *motB*, which are transporter encoding genes in *E. coli*.

## Figures and Tables

**Table 1:** Strains and plasmids used in this study.

Strains or Plasmids	Description	Reference or Source
*E. coli*		
APEC40	Avian pathogenic *E. coli* (APEC) 40, wild-type	Laboratory
APEC40/Δ*sdiA*	APEC40 *sdiA*-deletion mutant	This study
APEC40/pSTV28	APEC40 with the empty vector pSTV28, Cm^r^	This study
Δ*sdiA*/pSTV28	Mutant with the empty vector pSTV28, Cm^r^	This study
Δ*sdiA*/pC*sdiA*	Mutant with the complement plasmid pC*sdiA*, Cm^r^	This study
DH-5α	Clone host strain	Invitrogen
Plasmids		
PKD46	Expresses λ-Red recombinase Exo, Bet, and Gam, temperature sensitive, Ampr	[28]
PKD3	cat gene, template plasmid, Cmr Ampr	[28]
Pcp20	FLPλcI857 λ++pRRep(Ts), temperature sensitive, Cm^r^ Amp^r^	[28]
pSTV28	Low copy number cloning vector, Cm^r^	Takara
pC*sdiA*	pSTV28 with *sdiA* gene, Cm^r^	This study

**Table 2:** Oligonucleotide primers used in this study.

Primer Name	Oligonucleotide (5′–3′)
40-SdiA-F	ACTCTCAGGGGCGTTGCGGTTTACTATGCAGGATAAGGATTGTAGGCTGGAGCTGCTT
40-SdiA-R	CTGGCACGCAGGACAGAAAAGAGATCAAATTAAGCCAGTATGAATATCCTCCTTAGTTC
Check-SdiA-F	CTGGACGCCATTTCAAGC
Check-SdiA-R	TGCCGAGAATAATCAAGAAC
pC-*sdiA*-F	CCGGAATTCCTGCTTAACAAATCAGCAT
pC-*sdiA*-R	CCCAAGCTTTCAAATTAAGCCAGTAGCG
RT-*cbrA*-F	ACGGCTGGGAGCAACATA
RT-*cbrA*-R	GGCACCGCCAAAGATAAA
RT-*eamB*-F	GTGCTGGCAGGGATGAGT
RT-*eamB*-R	GTCCGTCTTCCTTTGTTGG
RT-*bdm*-F	TTGGTCAGCTCCATGAGA
RT-*bdm*-R	AATTCAACAGCAGAACCC
RT-*yafP*-F	ATGACCGCCAGTCAGCAT
RT-*yafP*-R	GCGTCCACCGTAAGTTCG
RT-*motB*-F	GGCTGCTTATTCACTTCCC
RT-*motB*-R	CCGACTTTATGACTGCGATG
RT-16s-F	TTTGAGTTCCCGGCC
RT-16s-R	CGGCCGCAAGGTTAA

**Table 3:** Susceptibility of *Escherichia coli* strains to various antibiotics.

Antibiotics	MIC (μg/mL) of Three *E. coli* Strains		
	APEC40	APEC40/Δ*sdiA*	40Δ*sdiA*/pC*sdiA*
Doxycycline	6.25	6.25	6.25
Gentamycin	0.5	0.5	0.5
Ofloxacin	3.125	3.125	3.125
Ciprofloxacin	3.125	3.125	3.125
Kanamycin	15.625	15.625	15.625
Erythromycin	56.8	56.8	56.8

MIC: the abbreviation for minimum inhibitory concentration.

## Data Availability

The data presented in this study are available on request from the corresponding author.
